# Soundscape Evaluation Comparison of Outdoor Activity Space Between Gated and Open Communities

**DOI:** 10.3389/fpsyg.2021.707477

**Published:** 2021-07-15

**Authors:** Peisheng Zhu, Xidong Liu, Xiaodong Lu, Fei Guo, Wanqi Tao, Xiaodi Han

**Affiliations:** School of Architecture and Fine Arts, Dalian University of Technology, Dalian, China

**Keywords:** soundscape evaluation, sound environment, residential area, gated community, open community, structural equation model

## Abstract

In communities, outdoor activity space is utilized most often by older adults and children, and the soundscape is very important for its quality. For different community planning modes, such as gated and open communities, focus should be on different soundscape enhancement strategies for outdoor spaces. In this paper, typical samples of activity spaces in a gated community and in an open community were used. The comparison was conducted through soundscape evaluation including an analysis of the dominance of various sound sources, noise annoyance, and the perceptual dimensions of soundscape. The results showed that noise annoyance in the gated community was significantly lower than in the open community, although the noise level was of no significance between the two communities. The community planning mode moderated the relationships among the soundscape perception parameters between the gated and open communities. To reduce noise annoyance in the gated communities, each sound source should be considered; in open communities, traffic noise only should be considered. In a gated community, adding natural sounds to reduce noise annoyance may be a feasible intervention; in an open community, this is not necessary. Besides, there was no relationship between noise annoyance and Eventfulness in an open community, indicating that noise annoyance was insufficient to explain the complex sound environment of the community. China’s community planning will gradually shift from a gated community to an open community, making the soundscape of outdoor activity spaces likely to change dramatically in the future. The findings will help urban designers and managers to adopt targeted strategies to improve the soundscape and quality of life of community-dwelling older adults and children.

## Introduction

Exposure to environmental noise can have negative effects on health (Passchier-Vermeer and Passchier, 2000; World Health Organization, 2011), such as the following: potentially developing cardiovascular diseases (Stansfeld and Matheson, 2003; Babisch et al., 2005; Basner et al., 2014), sleep disturbances (Öhrström and Skånberg, 2004; Basner and McGuire, 2018), cognitive impairment (Stansfeld et al., 2005; Ranft et al., 2009; Tzivian et al., 2016), psychological disorders (Tzivian et al., 2015), having negative effects on the auditory system (Muzet, 2007), and obesity (Christensen et al., 2015; Pyko et al., 2015). These potential negative outcomes are often related to older adults and children (Belojevic et al., 2008; Szalma and Hancock, 2011). Therefore, the sound environment is considered as a critical factor for creating a healthy city (Seidman and Standring, 2010). In China, when comparing various noise sources, a study showed that road traffic noise, as the main source of noise in urban areas, contributed to 61.2% of the noise (Zhang and Kang, 2007).

As an essential part of city planning, community is an important place for urban residents to rest and enjoy leisure. Residents, especially older adults and children, spend a lot of time outdoors to rest, play, talk, or engage in social activities; therefore, they need high-quality sound environments in such outdoor spaces (Brown et al., 2011). Accordingly, traffic noise control in communities has become one of the main objectives for different administrative sectors of the government. In February 2016, the Chinese government was actively promoting a construction model characterized by “narrow roads and dense road networks” and aimed to promote an open community design. In principle, it was decided that new communities should not be developed as gated communities, and the walls of existing gated communities should be gradually demolished. Such demolishing, accordingly, brought an immediate source of noise to the sound environment of many gated communities in China.

To try and reduce the impact of noise on residential buildings, many cities have carried out noise reduction plans by reducing road noise levels (European Parliament and Council, 2002; Ministry of Environmental Protection of the People’s Republic of China, 2008; Ng and Chau, 2014; Echevarria et al., 2016). However, the cited studies mainly focused on the change of noise level, giving less attention to perceptual changes. Moreover, many studies have focused on high-noise areas adjacent to roads, whereas less attention has been paid to low-noise areas, which are the main areas of daily activity for older adults and children.

Previous studies have shown that noise level reduction is not completely correspondent to acoustic comfort (Yang and Kang, 2005; Berglund et al., 2006; Meng and Kang, 2015; Gozalo et al., 2018), and sound energy is not highly correlated with soundscape perception (Jambrošić et al., 2013; Ma et al., 2018). Therefore, recently, some scholars have proposed that the quality of communities can be improved through soundscape construction (Vogiatzis and Remy, 2014; Gozalo and Morillas, 2017). Hong and Jeon (2015) developed a structural equation model (SEM) model to determine the relationship among various soundscape perception indicators in communities, proposing that traffic noise and human sound are the main sound sources that affect soundscape perception. Hao et al. (2016) found that the soundscape quality of traffic noise environments could be improved by the masking effects of birdsong. Dzhambov et al. (2018) proposed that a reasonable control of green space perception was conducive to reducing noise annoyance in communities. However, these studies did not distinguish the impact of the differences between diversified community planning modes.

Worldwide, there are two main community planning modes, gated and open communities. Generally speaking, gated communities in residential areas are defined by restricted access, designated parameters, walls or fences, controlled entrances intended to prevent penetration by non-residents, and the external surrounding streets are typically wide arterial roads (Davis, 1990; Roitman, 2010; Sun and Webster, 2019). Open communities are the opposite of gated communities, tending to have gridded road networks (Miao, 2003; Dong et al., 2019). Traffic demand, the distribution of traffic flow, the distances of buildings from the road, building formats, and even the activities and behaviors of people are different in these two modes (Miao, 2003). Thus, the sound environment may also differ.

**Figure 1:**
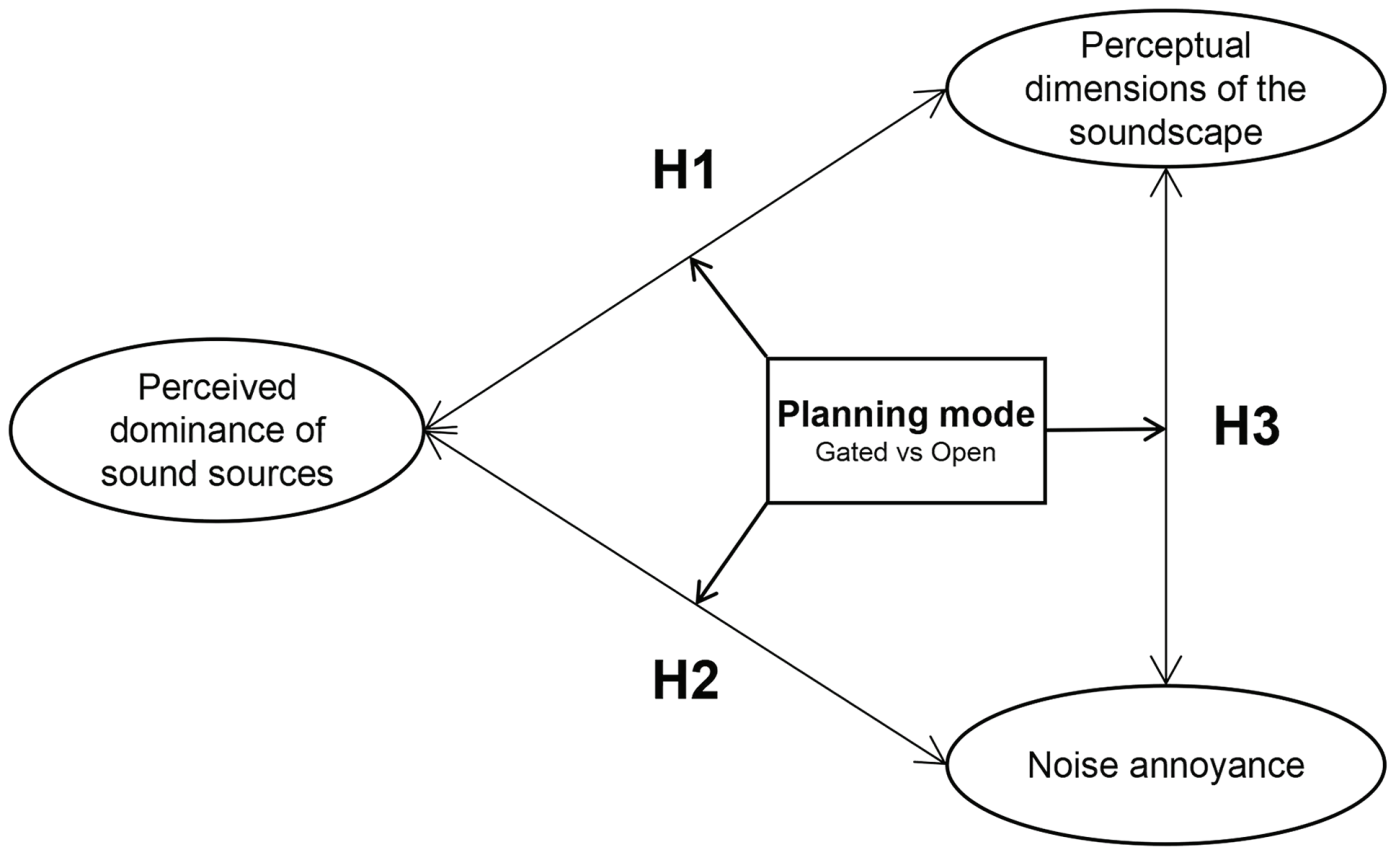
Conceptual framework of the study.

This study aimed to clarify the effect of community planning mode on soundscape perception parameters. Specifically, it aimed to determine (1) whether there are differences in the soundscape perception between different planning modes; (2) if yes, whether the planning mode affects the relationship among the soundscape evaluation parameters; and (3) if yes, which relationships among the soundscape perception parameters do significantly change between different planning modes? Moreover, in what way will the soundscape enhancement strategies differ? The results may help urban planners and city managers to choose appropriate strategies to improve the sound environment in communities for older adults and children. The selected soundscape evaluation parameters included the perceived dominance of sound sources, noise annoyance, and perceptual dimensions of the soundscape. Figure 1 presents the conceptual framework of this study. Based on the above review, the following hypotheses are developed.

*H1*: The relationship between the perceived dominance of the sound source and perceptual dimensions of the soundscape will differ between the gated and open communities.

*H2*: The relationship between the perceived dominance of the sound source and noise annoyance will differ between the gated and open communities.

*H3*: The relationship between noise annoyance and the perceptual dimensions of the soundscape will differ between the gated and open communities.

Owing to the following topics, this study was focused on the sound environment of low-noise areas: because the literature on high-noise areas (about 70 dBA) – which are mainly related to the presence of adjacent, noisy roads – has a plethora of findings (Allen et al., 2009). Most prior research has placed little concern on community planning modes; and low-noise areas (about 50 dBA) are the main activity areas for older adults and children. Two typical communities were selected to represent the gated and open community. Using questionnaires in a laboratory setting, a soundscape perception evaluation was conducted. A structural equation model was used to compare soundscape perception-related results of the gated and open communities.

## Materials and Methods

### Site Selection

**Figure 2:**
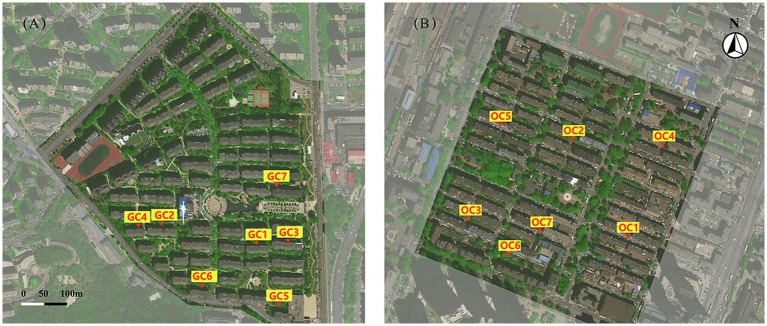
General community planning mode. **(A)** Xinghai Renjia Community (GC); **(B)** Xingshe Community (OC).

**Figure 3:**
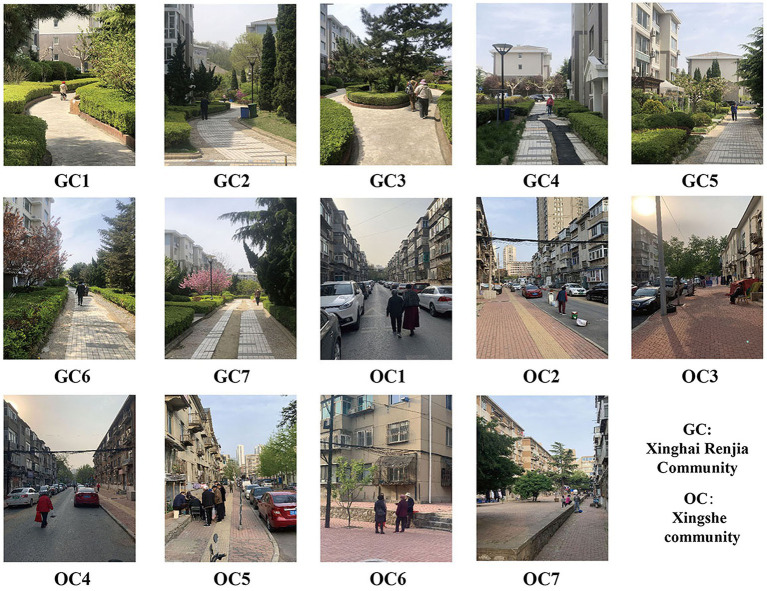
Photos of the sites in Xinghai Renjia Community (GC) and Xingshe community (OC) where the surveys were conducted.

The Xinghai Renjia Community and the Xingshe Community, both in Dalian City, China, were selected for comparative research (see Figure 2). The first was used to represent gated communities, whereas the latter to represent open communities. The Xinghai Renjia Community comprises an area of 0.21 km^2^ with 73 houses, and the Xingshe community comprises an area of 0.17 km^2^ with 55 houses; hence, they are similar in size. However, the planning modes of the two communities are different. The Xinghai Renjia community is a typical gated community with obvious boundaries, as the outside is surrounded by arterial roads and the inside exclusively by pedestrian roads, except for a single automobile road. The Xingshe community is a typical open community, with the outside being close to a city branch road, the inside comprising gridded streets, and automobiles being able to transit freely. The study identified seven locations in the gated community and the open community as measurement points, respectively, which were named as GC1–GC7 in the Xinghai Renjia community and OC1–OC7 in the Xingshe community, as shown in Figure 2. Photos of the *in-situ* are shown in Figure 3. These locations were evenly distributed to reflect the general situation within the community surrounded by buildings, in which there were a variety of noise sources such as traffic, people, and birds.

### Measurement of Sound Parameters

The measurement of the studied sound parameters was carried out in the two communities from 9:00–16:00 during a weekday in October. Noise level was measured using an NTI XL2 sound level meter including the A-weighted equivalent continuous sound pressure level (L_Aeq_), the C-weighted equivalent continuous sound pressure level (L_Ceq_), and statistical levels (L_A90_, L_A50_, and L_A10_).

To reflect the human binaural auditory system, binaural recordings were conducted, making it possible to reproduce the spatial characteristics of the recorded acoustic environments (Genuit and Fiebig, 2006; Semidor, 2006). Therefore, a 5-min audio recording of the sound environment at each location was taken using a binaural recording device BR2022, which was set at a sampling rate and bit depth of 48 kHz and 24 bits, respectively. Recording levels were calibrated by a sound calibrator B&K4231 (Jeon et al., 2018).

### Construction of the Questionnaire

Considering that the sound environment perceived by each person constantly changes in on-site soundscape evaluations, which makes it difficult to rule out the influence of occasional noise, the listening experiment was conducted in a laboratory by playing the recorded files obtained on the spot.

Participants were recruited to engage in the listening experiment and were required to complete a questionnaire, which contained three parts. The first part of the questionnaire was about perceived dominance of sound sources including three questions. The identification of sound sources is important for understanding soundscape perceptions because the different categories of sound sources provide more information on perceived soundscape quality (Nilsson et al., 2007; Hong and Jeon, 2015; Lu et al., 2020). Based on widely used variables present in soundscape studies (Hong and Jeon, 2015, 2017), the current study classified sound sources into: traffic noise, human sounds (i.e., sounds from human activities), and natural sounds. For each perceived dominance of sound source, the evaluation was made with a five-point Likert type scale (−2 = Do not hear at all, −1 = Hear slightly, 0 = Hear moderately, 1 = Hear a lot, and 2 = Hear predominantly). The left extremity of the bipolar scale was coded as “−2,” meaning that “Do not hear at all,” and its right extremity was coded as “2,” meaning that “Hear predominantly.”

The second part of the questionnaire concerned noise annoyance including one question. Noise annoyance mainly covers immediate behavioral effects and evaluative aspects related to noise (Guski et al., 1999) and is easily caused by major sound sources in urban areas such as road traffic and sounds from people in resident areas (Gidlöf-Gunnarsson and Öhrström, 2010; Di et al., 2012; Hong and Jeon, 2015; Gozalo and Morillas, 2017). Procedures for assessing noise annoyance have been investigated thoroughly (Levine, 1981; Fields et al., 1997). According to these procedures, noise annoyance evaluation was carried out using a five-point Likert language scale (−2, Not at all; −1, Slightly; 0, Moderately; 1, Very; and 2, Extremely).

The third part of the questionnaire investigated soundscape perception dimensions including eight questions. Axelsson et al. (2010) proposed a two-dimensional model of soundscape perceptions, which were defined by two orthogonal factors, Pleasantness and Eventfulness. These two factors have been commonly assessed in several soundscape studies (Kang and Zhang, 2010; Hong and Jeon, 2017; Jeon et al., 2018). In this study, and according to the standard emotional vocabulary provided by the Swedish Soundscape-Quality Protocol (Axelsson et al., 2010; Hong and Jeon, 2015, 2017; Aletta et al., 2016), eight adjectives were used for evaluating soundscape perception dimensions; they were Pleasant, Unpleasant, Chaotic, Calm, Eventful, Uneventful, Exciting, and Monotonous. For each adjective, the evaluation was made with a five-point Likert type scale (−2 = Strongly disagree, −1 = Slightly disagree, 0 = Neither agree nor disagree, 1 = Slightly agree, and 2 = Strongly agree). The left extremity of the bipolar scale was coded as “−2,” meaning that “Strongly disagree,” and its right extremity was coded as “2,” meaning that “2 = Strongly agree.”

### Procedure

In total, 30 participants (15 male and 15 female participants) were included in the listening experiments. They were all postgraduates, and their age distribution ranged from 22 to 29 years (mean = 26.0 years, SD = 2.0 years). Before participating in the experiment, aligned to prior research (Lu et al., 2020), all subjects were tested for air-conduction hearing threshold levels using a Madsen audiometer model ITERA. To ensure that the noise level meets the standard requirements (ISO/IEC IS 8253-1, 2010; International Organization for Standardization, 2010), the experiment was carried out in an audio-visual laboratory with certain sound insulation measures. According to the current standards (GBZ 49-2014; International Organization for Standardization, 2014), the results showed that all subjects had normal hearing.

It should be mentioned that actual dwellers in the same age group can be recruited for the laboratory experiments, which may have contributed to the reliability of the results. However, if the participants had not been of similar ages (Yang and Kang, 2005) or were, for example, older than 60 years, this may have caused large individual differences due to inconsistent hearing levels, thereby causing the conclusions to be unreliable. This study, therefore, recruited postgraduates, allowing for comparisons with other studies using young recruits.

The listening experiment was conducted using a Sennheiser HD-600 headphone, a Rane-HC4s corresponding power amplifier, and a B&K ZE-0948 audio interface. Given that the HD-600 headphone is a type of open-air headphone, it was easily affected by ambient noise; hence, based on prior research (Zhu et al., 2014; Jeon et al., 2018), the experiment was conducted in a semi-anechoic chamber. According to Zhu et al. (2014), to ensure that the noise level of the reproduced sound was exactly the same as that on the actual site, the binaural recording signals reproduced through a headphone that was placed on an artificial head and those recorded by the NTI XL2 sound level meter were compared.

**Figure 4:**
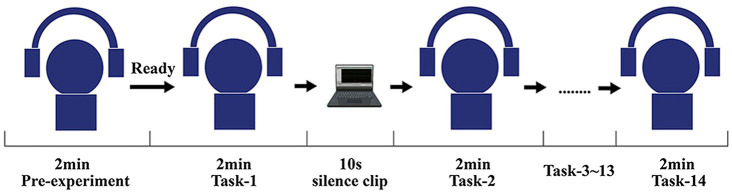
The experimental procedure.

Before the formal experiments, systematic training was provided to all participants to ensure that they understood the entire experiment process and could master the key points (Zhu et al., 2014). Based on a prior methodology (Jeon et al., 2018), the formal experiments were conducted as follows: each subject first listened to one audio excerpt (2 min) as a pre-exercise; then, 14 formal audio excerpts (2 min) were presented, in an irregular order, to every participant while they were concomitantly required to answer the questionnaires (see Figure 4). The final sample comprised 420 effectively responded questionnaires, 210 from each community.

The Wald-Wolfowitz runs test (Wald and Wolfowtiz, 1940; Bartels, 1982; Lu et al., 2019) conducted on 14 sets of data evaluations for each participant showed that the majority of the runs were insignificant, indicating that the datasets evaluated by each participant were independent. This inference can also be explained empirically. Each recording file is randomly recorded at different measuring points, and there are differences between each, which indicates that the soundscape evaluation is also independent to some extent. Thus, the sample size for both communities was 42, and for each community, the sample size was 210.

The sample size meets the requirements for statistical analyses. For a principal component analysis (PCA), the minimal adequate sample size should be five times the number of variables (O’Rourke and Hatcher, 2013). Regarding this study, the number of items is eight, which means that the sample size meets the requirements for each community (210 > 40). Additionally, the sample size for each community meets the requirements of SEM (210 > 200) (Kline and Little, 2011). Furthermore, a comparison between various sample sizes suggests that a sample size of 100–150 is generally acceptable for evaluating soundscapes in public spaces (Nilsson et al., 2007).

In addition, Table 1 shows the noise level results of the survey sites. Based on prior research (Gozalo et al., 2018; Lu et al., 2020), two calculations were conducted as follows: L_Ceq_–L_Aeq_ and L_A10_–L_A90_. *T*-tests were then conducted for noise level parameters, which showed no statistical difference in noise level indicators between the two communities, suggesting that the discussion below is based on the premise that sound energy is broadly the same in both communities.

**Table 1:** Noise level indicator results of survey sites in dB.

Survey sites		L_Aeq_	L_Ceq_	L_10_	L_50_	L_90_	L_Ceq_-L_Aeq_	L_10_-L_90_
Gated community	GC1	46.5	61.5	47.1	45.1	42.3	15	4.8
	GC2	47.6	63.9	50.1	45.6	42.1	16.3	8
	GC3	51.4	66.3	53	50.5	46.8	15	6.2
	GC4	50.4	67.9	52.8	49.8	44.4	17.6	8.4
	GC5	54	67	56.5	52.9	49.7	13	6.8
	GC6	53.2	66	55.3	52.3	49.5	12.8	5.8
	GC7	49.9	69.3	50.1	49	47.6	19.4	2.5
	Mean	50.4	66.0	52.1	49.3	46.1	15.6	6.1
	SD	2.5	2.4	3.0	2.8	2.9	2.2	1.9
Open community	OC1	49	65.8	50.4	47.8	46.4	16.8	4
	OC2	45.7	59.2	46.7	44.5	43.5	13.5	3.2
	OC3	53.6	70.5	56	52	48.7	16.9	7.3
	OC4	48.8	60.1	51.4	47.6	45	11.3	6.4
	OC5	53	66.7	56.2	51	44.3	13.6	11.9
	OC6	51.2	65.7	52.8	50.4	49	14.4	3.8
	OC7	49.5	61.6	49.3	47.6	46.8	13.1	2.5
	Mean	50.1	64.2	51.8	48.7	46.2	14.2	5.6
	SD	2.5	3.8	3.2	2.4	2.0	1.9	3.0

SD, standard deviation.

### Data Analysis

First, to determine whether there was a significant difference in the sound environment between the gated and open community, the mean values for the soundscape evaluation parameters were compared. Shapiro–Wilk was used to determine whether data were normally distributed. If yes, an independent samples *t*-test or independent samples Mann–Whitney *U* test was used. For the soundscape perceptual dimensions, the principal component scores were calculated by the adjectives and then compared between the two planning modes.

Subsequently, SEM was carried out in AMOS 21.0 to test whether the community planning mode moderated the soundscape. In the first step, exploratory factor analysis (EFA) and confirmatory factor analysis (CFA) confirmed that the two models showed acceptable goodness of fit. Following this, multi-group SEM was used to examine the potential moderating role of community planning modes, and whether the proposed mediation model showed significant differences between the gated and open communities. The regression coefficients in each group model were constrained, and the changes were evaluated to test the moderating effect of community planning modes.

The recommended cut-off values for goodness-of-fit were as follows: *χ*^2^/df < 5, root-mean-square error of approximation (RMSEA) < 0.1, comparative fit index (CFI) > 0.90, incremental fit index (IFI) > 0.90, and goodness-of-fit index (GFI) > 0.90 (Kline and Little, 2011).

## Results

### Comparison of Soundscape Evaluation

#### Comparison of Perceived Dominance of Sound Sources

**Figure 5:**
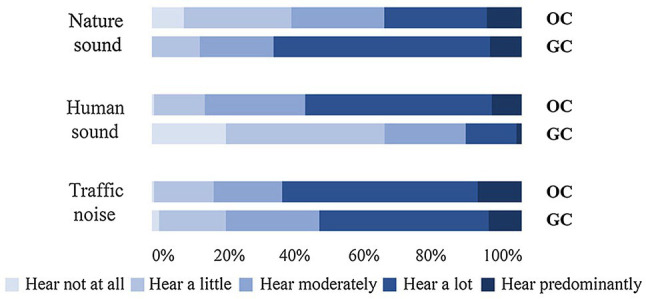
Results of the sound source evaluation stratified by the gated community and the open community and the three sound sources. GC, the gated community and OC, the open community.

Table 2 shows the descriptive statistics of the soundscape evaluation parameters. Figure 5 shows the evaluation results of perceived dominance for each sound source in the two communities. The dominance of traffic noise in the gated community (mean = 0.42, SD = 0.951) was lower than that in the open community (mean = 0.60, SD = 0.914). For traffic noise, the total response ratio for the options “Hear a lot” and “Hear predominantly” in the gated community (54.7%) was also larger than in the open community (64.8%). This was because, although the noise level of the two communities was basically the same, the traffic noise of the gated community comes from distant roads with reflected sounds accounting for a large proportion of the sound energy, and the overall fluctuation being small. In the open community, the traffic noise emanates from the nearby roads; therefore, the direct sound accounts for a large proportion of the sound energy, and the sound fluctuates greatly; these characteristics are obvious to our human perceptual capabilities.

**Table 2:** The descriptive statistics of the soundscape evaluation parameters.

Soundscape evaluation parameters	Observed variables	Total		Gated community		Open community		Skewness	Kurtosis
		*M*	*SD*	*M*	*SD*	*M*	*SD*		
Noise annoyance		−0.34	0.95	−0.52	0.93	−0.16	0.94	0.34	−0.45
Perceived dominance of sound sources	Traffic noise	0.51	0.94	0.42	0.95	0.60	0.91	−0.49	−0.48
	Human sound	−0.07	1.10	−0.66	1.00	0.52	0.85	−0.11	−0.96
	Natural sound	0.32	1.04	0.63	0.82	0.00	1.14	−0.41	−0.66
Soundscape perception dimensions	Pleasant	−0.04	0.85	0.12	0.86	−0.20	0.81	−0.06	−0.53
	Unpleasant	0.13	0.86	0.26	0.85	0.00	0.86	−0.21	−0.50
	Chaotic	−0.07	1.07	0.20	1.05	−0.33	1.03	0.20	−1.12
	Calm	−0.26	1.04	0.02	1.05	−0.54	0.96	0.26	−0.94
	Eventful	0.24	1.06	−0.14	1.04	0.62	0.94	−0.30	−1.02
	Uneventful	0.51	1.02	0.18	1.06	0.84	0.87	−0.60	−0.36
	Monotonous	0.44	0.94	0.18	0.95	0.71	0.85	−0.50	−0.42
	Exciting	−0.40	0.91	−0.60	0.87	−0.20	0.90	0.00	−0.56

Total (*N* = 420), gated community (*n* = 210), and open community (*n* = 210).

The dominance of human sound in the gated community (mean = −0.66, SD = 0.995) was much lower than in the open community (mean = 0.52, SD = 0.848). For human sound, the total response ratio for “Hear a lot” and “Hear predominantly” was also diminished in the gated community (15.2%) compared with the open community (58.6%). This is because different planning modes bring forth different architectural functions and crowd activities. Compared with the gated community, the open community has more commercial facilities facing the street; therefore, there are more human activities that occur near or in the streets. Hence, there were more natural human sounds that could be heard at the open community test site.

The dominance of natural sound in the gated community (mean = 0.63, SD = 0.816) was much higher than in the open community (mean = 0.00, SD = 1.139). The total response ratio for “Hear a lot” and “Hear predominantly” was also higher in the gated community (67.2%) than in the open community (37.1%). This is because, compared with the open community, the ecology in the gated community tends to be better and, therefore, more conducive to birds settling there. Hence, more natural sounds were heard at the gated community test site.

The Mann–Whitney *U* test showed that there were significant differences in the perceived dominance of all three sound sources in the two communities.

#### Comparison for Noise Annoyance

**Figure 6:**
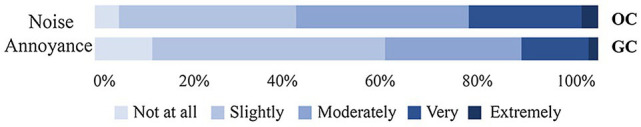
Results of the evaluation of noise annoyance of the gated community and the open community. GC, the gated community and OC, the open community.

As can be seen from Table 2 and Figure 6, noise annoyance in the gated community (mean = −0.52, SD = 0.93) was significantly lower than in the open community (mean = −0.16, SD = 0.94). The total response ratio for “Very” and “Extremely” (15.2%) was also diminished in the gated community compared with that in the open community (25.7%). The Mann–Whitney *U* test showed that the noise annoyance in the two communities differed significantly.

#### Comparison of the Soundscape Perception Dimensions

To obtain the differences in soundscape perception by community planning mode, the data matrixes of the 420 individual responses to the third section of the questionnaire were created. To determine the optimized orthogonal components, varimax rotation was applied. Following the Kaiser criterion (Kaiser, 1960), two components with eigenvalues larger than one were obtained for the data, and they explained 68.91% of the total variance. Based on prior research, the minimum acceptable value is 60% (Reyment and Jvreskog, 1993).

Two main dimensions, labeled as Pleasantness and Eventfulness were extracted, concurring with prior research (Axelsson et al., 2010). The Pleasantness dimension contained the Pleasant, Unpleasant, Chaotic, and Calm adjectives and explained 35.07% of the total variance. The Eventfulness dimension contained the Eventful, Uneventful, Monotonous, and Exciting adjectives and explained 33.84% of the total variance. To analyze the data from each subject, component scores of Pleasantness and Eventfulness were calculated using the regression method – as described in prior research (Guski et al., 1999). The Mann–Whitney *U* test showed that there were significant differences in the soundscape perception dimension between the two communities as can be seen in Table 2.

An EFA using PCA was applied to extract the soundscape perception dimensions. By examining the reliability and validity of the evaluations in the PCA solution, the study reversed the direction of the evaluations of four adjectives: Unpleasant, Chaotic, Uneventful, and Monotonous, ensuring that they maintained a consistent score. Results showed that the respective Cronbach’s alpha coefficient for the PCA was 0.849; hence, it was over 0.80, suggesting good reliability (Lance et al., 2006). The Kaiser–Meyer–Olkin (KMO) measure of sampling adequacy was 0.787; hence, it was over 0.70, confirming the validity of the questionnaire. The Bartlett’s spherical test results (*p* = 0.000 < 0.01) were also meaningful (Hair et al., 2010).

### Reliability and Validity of Research Constructs Using SEM

**Figure 7:**
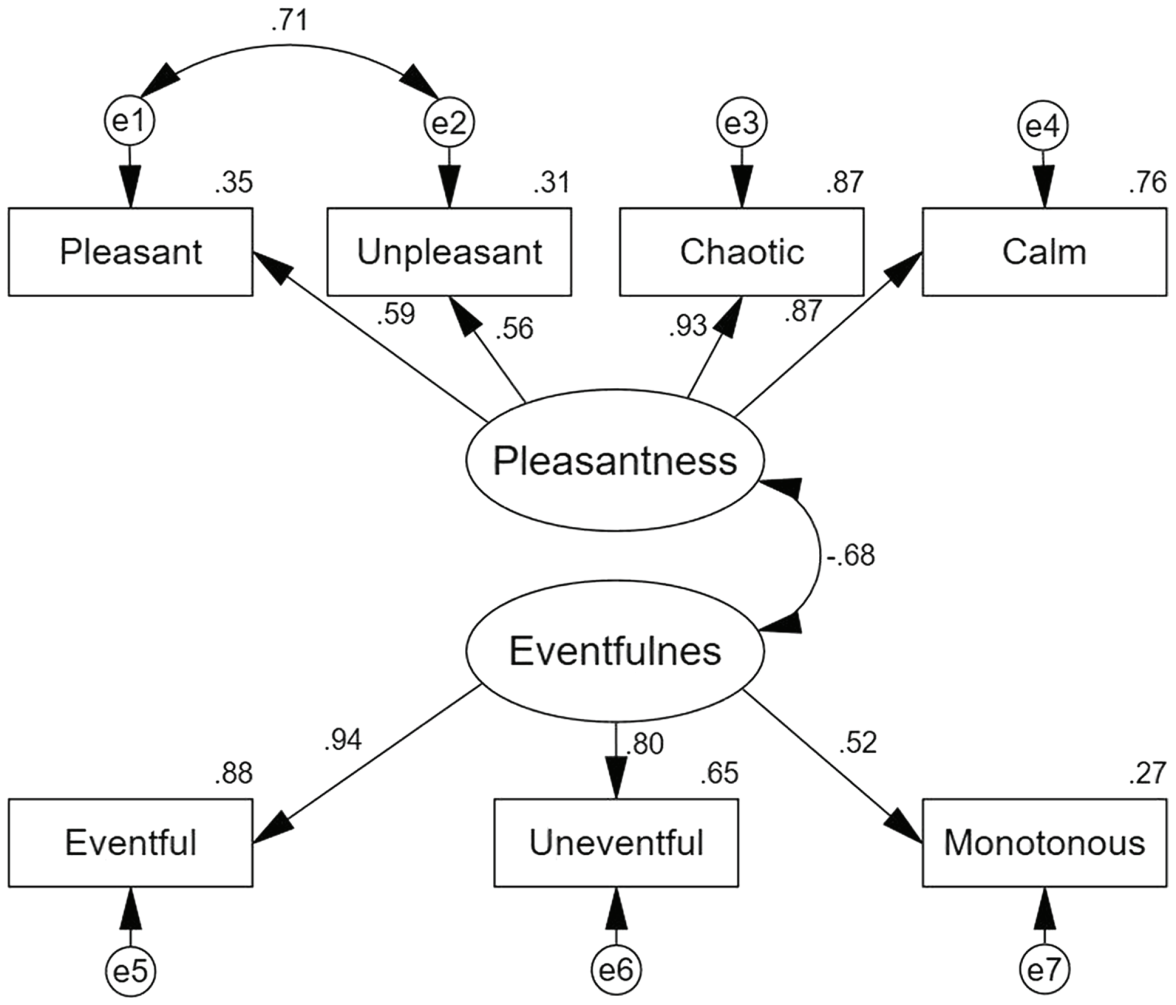
Schematic description of the results for the CFA of the two first order factors (*N* = 420). All coefficients are significant at the *p* < 0.05 level.

The measurement model was tested using CFA (see Figure 7). The item loading was defined as the ratio between the item (question-statement) and the construct. The item loadings needed to be equal to or greater than 0.50 based on prior research (Kock, 2015). As the loading for the exciting item was 0.45–0.50, it was excluded from the study. All other item loadings were above 0.50, serving as validation parameters of the CFA (Kock, 2015).

Convergent validity and discriminant validity were then tested. To ensure scale reliability and internal consistency, Cronbach’s alpha was applied and needed to be at least 0.70 (Peterson, 1994). The composite reliability analysis showed that the constructs were mutually interchangeable. As all the constructs exhibited an internal consistency, reliability was higher than the set target of >0.7 based on prior research (Hair et al., 2013). The average variance extracted (AVE) was defined as the proportion of variance in the items explained by the relevant construct. To ensure validity for the scale, the recommended AVE threshold was 0.50 (Kock, 2015), as an AVE of 0.50 represents that, on average, a construct can explain about 50% of the variance of its indicators. Table 3 displays Cronbach’s alpha, the AVE, and critical ratio, all of which being satisfactory.

**Table 3:** Results of confirmatory factor analysis (CFA) for scale reliability and construct validity (*N* = 420).

Latent variable	Observed variable	Reliability (Cronbach’s *α*)	Factor loadings	CR	AVE
Pleasantness	Pleasant	0.861	0.591	0.83	0.57
	Unpleasant		0.556		
	Chaotic		0.931		
	Calm		0.869		
Eventfulness	Monotonous	0.804	0.524	0.81	0.60
	Uneventful		0.804		
	Eventful		0.938		

AVE, average variance extracted; and CR, critical ratio.

Modification indices were applied though added error covariance between Pleasant and Unpleasant according to empirical rationales (Kline and Little, 2011). The results demonstrated acceptable model fit indices: *χ*^2^/df = 3.93, CFI = 0.98, IFI = 0.98, GFI = 0.97, and RMSEA = 0.08. The skewness and kurtosis values, which were within the range of −1.12 and 0.26, indicated normally distributed variables – based on prior research (Smedema et al., 2010).

### Multi-Group Analysis in the SEM

To test the moderating effect of community planning mode, group differences between gated and open communities were determined using multi-group analysis of SEM. The unconstrained structural model, which allowed for the structural paths to vary across community planning modes, was compared with the constrained structural model, which constrained the regression coefficients to be equal between the communities. The results showed that the unconstrained model (*χ*^2^ = 254.42, df = 66) and constrained model (*χ*^2^ = 66, df = 77) had significant differences (*p* < 0.01), suggesting that the community planning mode played a moderating role in the relationship among the different soundscape parameters.

The factor loadings of all items for two latent variables ranged from 0.43 to 0.95 in the two SEMs, suggesting good values for all variables (Kline and Little, 2011). Table 4 shows the fitness indicators in the SEMs for the unconstrained and constrained model. The fitness index of the grouping model conformed to the recommended value, indicating that the theoretical model was valid.

**Table 4:** Fitness indicators of structural equation models for the gated and open communities.

Fit indicators	*χ*^2^/df	RMSEA	CFI	IFI	GFI
Unconstrained model	3.85	0.08	0.91	0.91	0.90
Constrained model	3.56	0.08	0.91	0.91	0.90

RMSEA, root-mean-square error of approximation; CFI, comparative fit index; IFI, incremental fit index; and GFI, goodness-of-fit index.

**Figure 8:**
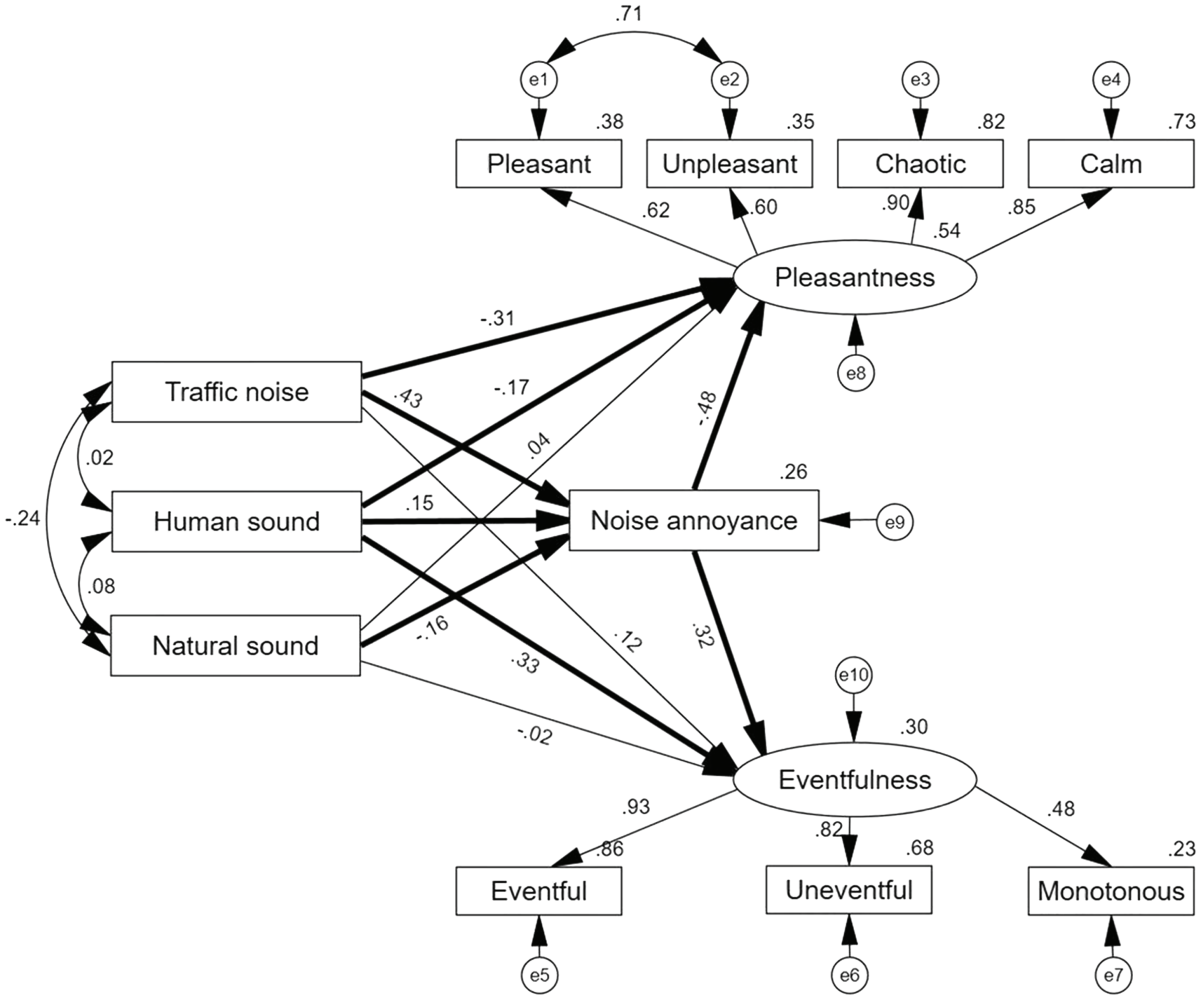
Results of the multigroup analysis in the structural equation model (SEM) for the gated community (*N* = 210). The presented coefficients are standardized. e, error term, residue term; bold line, significant; and thin line, non-significant.

**Figure 9:**
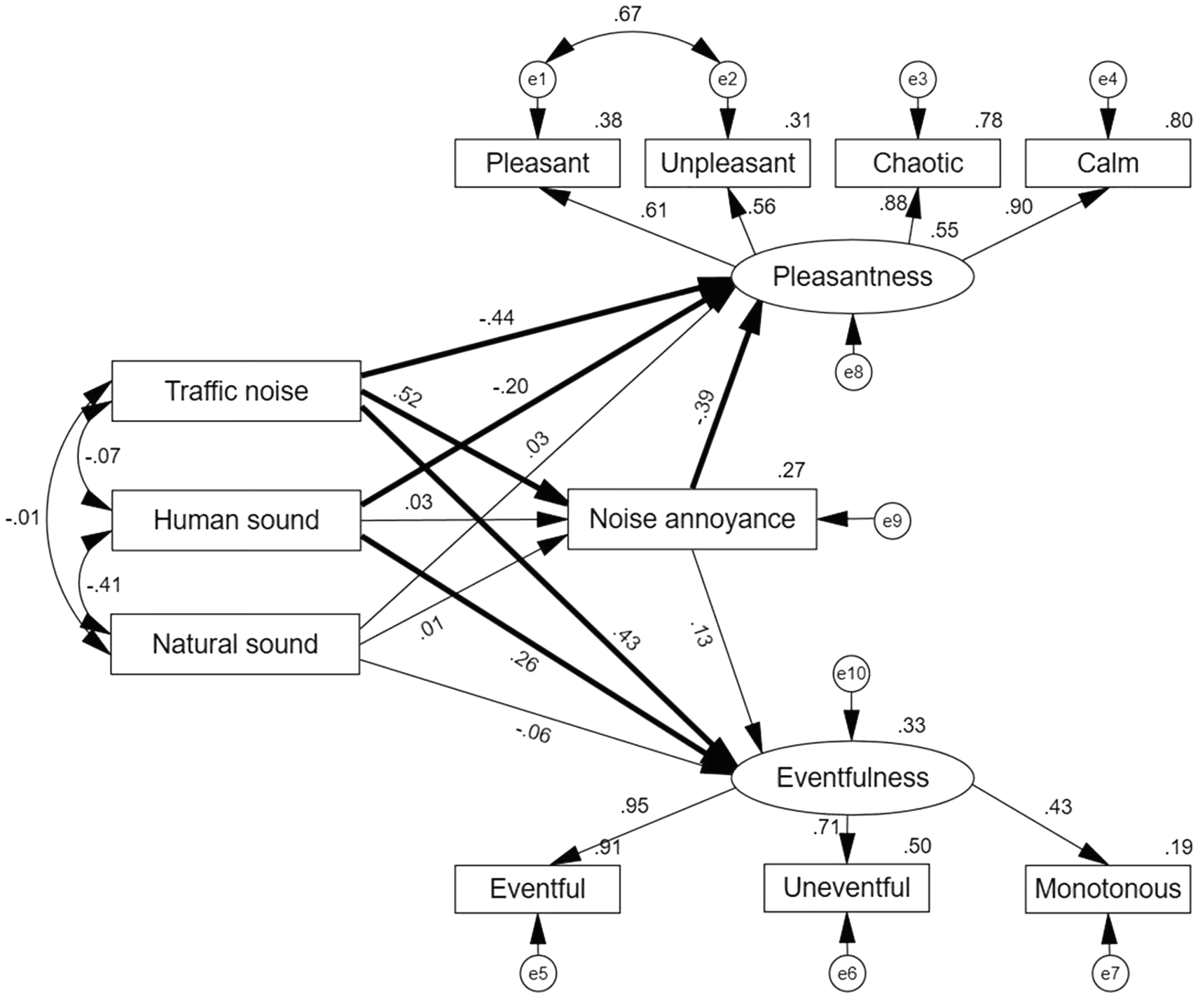
Multigroup analysis in the SEM for the open community (*N* = 210). The presented coefficients are standardized. e, error term, residue term; bold line, significant; and thin line, non-significant.

As can be seen from Figures 8, 9, the two SEMs revealed that traffic noise and human sound had a significantly negative (*p* < 0.05) effect on Pleasantness and a positive effect (*p* < 0.05) on Eventfulness, in both gated and open communities. Moreover, traffic noise was positively associated with noise annoyance in both communities. Natural sound had non-significant (*p* > 0.05) effects on Pleasantness and Eventfulness. Further, noise annoyance was negatively associated with Pleasantness and positively associated with Eventfulness. Moreover, in the gated community, the dominance of the three sound sources was significantly related to noise annoyance; in the open community, only the dominance of traffic noise was significantly related to noise annoyance. Namely, to reduce noise annoyance, the gated community needs to have every type of sound source in it targeted and dealt with, while the open community requires only traffic noise to be diminished.

To clarify which relationships among the soundscape perception parameters have changed between the gated and open communities, path-by-path comparisons were conducted using critical ratios for differences (CRD); this method served to allow for examining the existing differences in each structural path across the two groups. If the CRD between the two groups is between ±1.96 using pairwise parameter comparison (Arbuckle, 2011; Byrne, 2013), then no difference existed between the two groups, otherwise, a significant difference existed.

As presented in Table 5, the CRD test showed that the paths from traffic noise to Eventfulness were statistically and significantly different between the two groups (CRD = 2.75, *p* < 0.05); specifically, traffic noise positively predicted Eventfulness in the open community (*β* = 0.43, *p* > 0.05), but showed no significant effect in the gated community (*β* = 0.12, *p* > 0.05). This means that greater emphasis should be placed on measures to reduce traffic noise fluctuations in the open community compared with the gated community. Also, it proved that the general research hypothesis, H1, was statistically significant.

**Table 5:** Path coefficients for the relationships among the soundscape perception parameters and comparison between the gated and open community.

Path of regression	Gated community	Open community	CRD
Traffic noise → Pleasantness	−0.17^***^(−0.31)	−0.24^***^(−0.44)	−1.13
Human sound → Pleasantness	−0.09^**^(−0.17)	−0.11^**^(−0.2)	−0.48
Natural sound → Pleasantness	0.02(0.04)	0.01(0.03)	−0.22
**Traffic noise → Eventfulness**	**0.12(0.12)**	0.42^***^(0.43)	**2.75**
Human sound → Eventfulness	0.32^***^(0.33)	0.28^***^(0.26)	−0.39
Natural sound → Eventfulness	−0.02(−0.02)	−0.04(−0.06)	−0.26
Traffic noise → Noise annoyance	0.42^***^(0.43)	0.54^***^(0.52)	1.42
Human sound → Noise annoyance	0.14^**^(0.15)	0.03(0.03)	−1.26
**Natural sound →Noise annoyance**	−0.18^**^(−0.16)	**0.01(0.01)**	**2.12**
Noise annoyance → Pleasantness	−0.28^***^(−0.48)	−0.2^***^(−0.39)	1.27
**Noise annoyance → Eventfulness**	0.34^***^(0.32)	**0.12(0.13)**	**−2.03**

CRD, critical ratios for differences between parameters. The non-standardized path estimates are reported outside brackets, and the standardized path estimates are reported in brackets. Bold text represents the significantly different paths between the gated and open communities.

** *p* < 0.01;

*** *p* < 0.001.

Further, the paths from natural sound to noise annoyance were statistically and significantly different between the two groups (CRD = 2.12, *p* < 0.05), which proved that the general research hypothesis, H2, was statistically significant. In the gated community, natural sound showed a negative effect on noise annoyance (*β* = −0.16, *p* < 0.001), and the opposite effect occurred in the open community (*β* = 0.01, *p* > 0.05). Hence, to reduce noise annoyance, stakeholders should add natural sounds to a gated community, whereas this is not necessary in an open community.

The paths from noise annoyance to Eventfulness (CRD = −2.03, *p* < 0.05) also showed a significant difference, which proved that the general research hypothesis, H3, was statistically significant. In the gated community, noise annoyance showed a positive effect on Eventfulness (*β* = 0.32, *p* < 0.001), while there was no such significant relationship in the open community. Therefore, noise annoyance could not, as a unique evaluation index, explain the complex sound environment in communities.

These results demonstrated that the community planning mode moderated the relationships among the soundscape perception parameters differently between the gated and open community. No significant group differences were found in other structural paths.

## Discussion

Although there were no significant differences in noise level indicators, the three soundscape perception variables showed significant differences between the gated and open communities, which further proves that the contribution of sound sources to soundscape quality might be different in different scenarios (Hong and Jeon, 2015). The dominance of traffic noise and human sounds in the gated community were lower than their dominance in the open community. The dominance of natural sounds in the gated community was higher than in the open community, and noise annoyance in the gated community was much lower than in the open community. Furthermore, the scores for the Pleasantness and Eventfulness dimensions of soundscape perception also significantly differed between the two communities’ environments.

The study confirmed that the community planning mode moderated the relationships among the soundscape perception parameters between the gated and open community. This means that for different residential planning modes, the corresponding sound environment improvement strategies should be different. However, previous studies on residential areas often ignored the influence of the planning mode and only proposed the need to control traffic noise (Hong and Jeon, 2015). In gated communities, reducing noise annoyance may encompass the consideration of each sound source, be it either to increase or decrease their sounds; in open communities, only traffic noise may need to be considered for such aims. Specifically, a comparison of the path analysis of the two communities showed a significant difference in the relationship between natural sound and noise annoyance in both communities; namely, in gated communities, attempts can be made to reduce noise annoyance by adding natural sound. Meanwhile, in open communities, there may be no such requirement to increase natural sounds. This is different from the study of Hao et al. (2016), which found that annoyance and Pleasantness can be altered by increasing the volume of birdsong in the low noise area (<52.5 dB), regardless of the planning mode. This may be caused by the difference in experimental methods, that is, our research considered the influence of reflections from surrounding buildings, while the study by Hao et al. (2016) did not.

There were also significant differences between noise annoyance and Eventfulness in the two communities. Noise annoyance had a positive effect on Eventfulness in the gated community, but a non-significant correlation in the open community; this indicated that for a community with complex internal environments, only considering noise annoyance may be insufficient to improve the soundscape. We suggest that future research should analyze noise fluctuation indexes.

Although the findings of this study were derived from data collected from the activity spaces of older adults and children in two typical communities, ensuring that the findings are applicable to many communities in China, influencing factors such as urban traffic flow, building layout, and site facilities were not carefully analyzed. Further, the major conclusions of this study were derived from a laboratorial sound playback; in a real environment, vision also has an impact on hearing and may change the outcomes. In the future, related research should focus on practical soundscape design.

## Conclusion

This study selected the main outdoor activity spaces (about 50 dBA) utilized by older adults and children in two typical communities in China as the measurement samples. The study was based on the premise that the noise level indicators are basically the same in both communities. Comparisons were then made for the soundscape perception between the gated and open communities. The conclusions were as follows:

1. The three soundscape perception variables showed significant differences between the gated and open communities, although the noise level was of no significance. It could be further explained that even if the objective characteristics of the sound environments are similar, there are still differences in soundscape perception, which is mainly due to the different modes of communities resulting in different content of the sound environment.
2. The community planning mode moderated the relationships among the soundscape perception parameters between the gated and open communities. To reduce noise annoyance in the gated communities, each sound source should be considered, particularly the addition of natural sounds; in open communities, only traffic noise should be considered.
3. There was no relationship between noise annoyance and Eventfulness in an open community, indicating that noise annoyance was insufficient to explain the complex sound environment of the community.

These findings will help urban designers and managers to adopt targeted strategies to improve the soundscape and quality of life of community dwellers. There are however limitations to this study. For example, the research conclusions (e.g., open communities do not need to increase natural sound), apply mainly to low-noise areas (50 dBA) in communities. In high-noise areas, the conclusions may be different. For example, Lu et al. (2020) believe that it is more effective to add natural sound in a high-noise area adjacent to a road than in a low-noise area, no matter what the planning mode. Therefore, in future studies, additional experiments should be conducted in high-noise areas to find possible differences between the two planning modes. Moreover, the communities selected for this study were all multi-story buildings. The sound environment perception of the communities with high-rise buildings may be different from those with multi-story buildings, considering that the sound environment perception will be affected by the building form (Echevarria et al., 2016). Therefore, in future studies by the present authors, experimental samples will be carried out on communities with various building forms.

## Data Availability Statement

The data used to support the findings of this study are available from the corresponding author upon request.

## Ethics Statement

Ethical review and approval was not required for the study on human participants in accordance with the local legislation and institutional requirements. The patients/participants provided their written informed consent to participate in this study.

## Author Contributions

XLu, PZ, and XLiu: research idea and study design. XLiu, WT,and XH: data collection. PZ, XLiu, XLu, and FG: data analysis and paper writing. PZ and XLu: supervision, project administration, and funding acquisition. All authors contributed to the article and approved the submitted version.

### Conflict of Interest

The authors declare that the research was conducted in the absence of any commercial or financial relationships that could be construed as a potential conflict of interest.
